# Confidence in providing methadone maintenance treatment of primary care providers in Vietnam

**DOI:** 10.1186/s13722-023-00419-5

**Published:** 2024-05-22

**Authors:** Bich Diep Nguyen, Li Li, Chunqing Lin, Thu Trang Nguyen, Steven Shoptaw, Minh Giang Le

**Affiliations:** 1 Center for Training and Research On Substance Abuse, HIV/AIDS, Hanoi Medical University, No 1, Ton That Tung Street, Dong Da District, Hanoi, Vietnam; 2 Department of Psychiatry and Biobehavioral Sciences, Semel Institute for Neuroscience & Human Behavior, University of California, Los Angeles, CA USA; 3 Department of Family Medicine, University of California, Los Angeles, CA USA; 4 Department of Epidemiology, School of Preventive Medicine and Public Health, Hanoi Medical University, Hanoi, Vietnam

**Keywords:** Methadone maintenance treatment, Confidence, Primary care providers, Vietnam

## Abstract

**Background:**

Delivering methadone treatment in community health facilities by primary care providers is a task-shifting strategy to expand access to drug use treatment, especially in rural mountainous areas. This study aims to investigate factors related to confidence in providing methadone treatment among primary care providers in Vietnam to inform good practice development.

**Methods:**

We conducted a cross-sectional survey with 276 primary care providers who were physicians, physician assistants, nurses, pharmacists or dispensing staff from 67 communes in a mountainous province in Northern Vietnam. Using self-report scales, we measured providers’ confidence in providing methadone treatment, beliefs in harm reduction, perceived work-related support, perceived stigma and risk in working with drug-using patients, and empathy towards this population. We used multiple linear regression analyses to explore factors associated with providers’ confidence in providing methadone treatment in the whole sample and to compare two groups of providers who did and did not have experience providing methadone. Potential associated factors were measured at facility and provider levels.

**Result:**

114 (41.3%) participants had previously experience in providing methadone treatment. Providers with methadone treatment experiences had higher confidence in and more accurate knowledge of methadone treatment, perceived less stigma of working with drug-using patients, and reported more work-related support than those without experiences. Higher medical education is associated with lower confidence in providing methadone treatment among providers without methadone experiences, but higher confidence among providers with methadone experiences. Better methadone knowledge was associated with greater confidence in providing methadone treatment among inexperienced providers but not among those with experiences. Receiving work-related support was associated with greater confidence in providing treatment in both groups, regardless of their past methadone experiences.

**Conclusion:**

In rural provinces where methadone treatment has been expanded to primary care clinics, interventions to improve primary care providers’ confidence should benefit professionals with diverse experiences in providing methadone treatment. Continued training and support at work for providers is essential to ensuring quality in decentralized methadone treatment.

## Introduction

Methadone maintenance treatment saves lives and improves the quality of life for people with opioid use disorder [14, 21, 38]. However, its global coverage is low, making its positive impact suboptimal [17, 37]. The greatest barrier to access and retention in methadone treatment is the daily observed dosage requirement still enforced in many countries, making it particularly challenging for people living in mountainous and rural areas with transportation difficulties [6, 30].

To address this barrier, several countries have shifted methadone treatment from specialized clinics to community health facilities to bring the treatment closer to people in need [2, 31, 41]. Challenges associated with this decentralization of methadone treatment include hesitancy and low capacity of primary care providers (PCP) at community health facilities, as drug use treatment is beyond their scope of practice [8, 20, 25]. The more PCPs are confident in providing methadone treatment, the more they are willing to take on new responsibilities and the better services they provide [13, 16, 32, 35]. Hence, understanding what relates to the confidence of PCPs in providing methadone treatment at community health facilities is crucial to the decentralization program.

Methadone maintenance treatment (MMT) in Vietnam has expanded nationwide since 2010 thanks to its efficacy in eradicating injection-driven HIV transmission[11]. Currently, the country has 341 specialized clinics in all 63 provinces, serving 55,000 patients [29]. A standard specialized clinic, comprised of two physicians, two counselors, two pharmacists or medication dispensing staff, and one administrative staff, is supposed to provide daily methadone to 250 patients [26]. To deal with travel difficulties in rural and mountainous areas, in 2015, Vietnam decentralized methadone treatment from these clinics to commune health centers (CHC). CHCs function as the dispensing sites of the main clinic in each district or province. Thus, methadone patients receive their daily methadone doses at the CHC in their local community, with periodic medical check-ups and counselling sessions at the main clinics [27]. In communes that are far from the main clinics, physicians/physician assistants and counselors can perform the periodic medical check-ups and provide counseling for patients. Most CHCs have no physicians on staff, rather only physician assistants with 2 or 3 years of medical education. To provide methadone treatment, each CHC has at least three part-time staff members, including one physician or physician assistant who clinically supervises the treatment, one counselor, and one staff member that dispenses medication. Providers were assigned by the local leaders (i.e., CHC and district health centers) to participate in the methadone program. Providers receive accreditation training comprised of one-week didactic lectures and a one-week practicum in an established MMT clinic before performing MMT tasks, and potentially advanced or refresher courses related to substance use disorders (SUD) treatment later [26].

By the end of 2021, 24/63 provinces opened methadone dispensing sites at 232 CHCs [29]. Most dispensing sites opened in mountainous provinces where patients encounter the greatest travel difficulties to receive daily methadone doses. Dien Bien is among the first provinces to implement both decentralized antiretroviral treatment (ART) and methadone treatment. By the end of 2018, the province provided methadone treatment and ART in 34 and 68 of its 130 communes, respectively. CHC in Dien Bien served 45% of all patients (about 2500) in the provincial program [7].

Vietnam, with its nationwide primary health care system, would be ideal to study methadone decentralization. The country has about 12,000 CHCs that are responsible for primary health care for people in the local community [28]. PCPs’ confidence in providing methadone treatment is important to ensuring treatment quality (for providers who are already involved in the program) and to expanding the coverage (for those who have not been previously involved) of the decentralization program. We hypothesize that PCPs having previously experience in methadone treatment would have higher confidence in providing the treatment. We explored the factors associated with confidence levels of PCPs with and without methadone experience. Our findings would inform intervention approaches to improve the quality of methadone decentralization in primary care settings.

## Methods

### Study participants

We conducted a cross-sectional survey among PCPs in Dien Bien in November and December 2019. We included all 29 CHCs that provided methadone treatment and randomly selected 38/101 CHCs without this service. We enrolled in total 67 CHCs in 9 districts. In the CHCs that provided methadone treatment, we recruited those who had been working in the local methadone program, including program managers and clinical providers (physicians, counselors, pharmacists and/or medication dispensing staff). In the CHCs that provided no methadone treatment, potential participants were physicians/physician assistants, nurses, pharmacists, or medication dispensing staff currently working at the CHC. All eligible PCPs in the selected CHCs were invited to participate in the study. Potential participants were informed about the study objectives, procedures, risks, and benefits before giving their verbal consent to participate in the study.

### Data collection

Study participants completed an assessment on a tablet in a private office at their workplaces. Participants read the on-screen questions and entered their responses directly into a computerized database. Our trained staff were available on site to provide instructions on how to use the online system and clarify the survey questions as needed. All questions were in Vietnamese. Each assessment took between 45 and 60 min to complete. Participants received 100,000 VND (approximately 5 USD) after completing the assessment compensation for their time.

### Measures

#### Outcome

*Confidence in providing methadone treatment* was measured with a 15-item scale (Cronbach’s alpha 0.93). We adapted this scale from a standard generalized self-efficacy scale [36] and the national treatment guidelines at methadone dispensing sites [27]. The questions asked about how confident PCPs were in working with methadone patients and completing tasks in methadone treatment. For example, questions asked PCPs to report if they were confident treating patients in different treatment phases or managing methadone storage and dosage. Responses included 1 = “not true”, 2 =  “hardly true”, 3 = “moderately true” and 4 = “totally true”. All scores were summed, 

#### Predictors

*We measured methadone treatment knowledge* with 20 true/false questions adapted from Caplehorn et al. [5] and the Vietnam national training materials for methadone providers [26]. The questions covered methadone pharmacokinetics (e.g., methadone can damage the liver), treatment requirements (e.g. methadone patients need to visit the dispensing clinic weekly), and effectiveness (e.g. methadone can elicit the same euphoria as heroin does). We coded false and true answers as 0 and 1, respectively. All scores were summed, and a higher total score indicated better methadone knowledge.

*Perceived work-related support* was measured with a 3-item scale (Cronbach’s alpha 0.84). This scale was a sub-scale of the Drug problems perceptions questionnaire [40]. Items asked whether PCPs perceived technical support while working with people who use drugs (PWUD). Responses ranged from 1 = "strongly agree" to 7 =  “strongly disagree”. All scores were reverse-coded, then summed. A higher total score indicated more perceived work-related support.

*Belief in harm reduction* approach of substance use and methadone treatment was measured with 4 items about harm reduction perspectives of substance abuse (Cronbach’s alpha 0.66) [23]. These items are: (1) Substance abuse is a chronic disease, (2) Relapsing individuals should be allowed to remain in MMT for substance abuse, (3) All opioid users wanting MMT should receive them, and (4) Reducing the harmful consequences of substance abuse is as important as achieving abstinence. Responses ranged from 1 = “strongly agree” to 4 = “strongly disagree”. All scores were reverse-coded, then summed. A higher total score indicated a stronger belief in harm reduction.

*Empathy towards PWUD* was measured with a 20-item scale (Cronbach’s alpha 0.86). We adapted this scale from the Jefferson scale of empathy [12]. The questions measured PCPs’ empathy towards PWUD and their empathy skills in working with PWUD. Response categories ranged from 1 = “strongly agree” to 5 = “strongly disagree”. After reverse-coding some items, all scores were summed, and a higher total score indicated more empathy.

*Perceived stigma of working with PWUD* was measured with a 6-item scale (Cronbach’s alpha 0.88) which was used in a previous study in China [18]. The items were about PCPs’ experiences of stigma and discrimination related to their work with PWUD. Response categories ranged from 1 = “strongly agree” to 5 = “strongly disagree”. All scores were summed, and a higher summary score indicated a higher level of their perceived stigma.

*We measured perceived risk in working with PWUD* using four questions about feelings of personal safety and risks of becoming infected with HIV, tuberculosis, and hepatitis when working with PWUD (Cronbach’s alpha 0.84) [3]. Response categories ranged from 1 = “strongly agree” to 5 = “strongly disagree”. All items were reverse-coded, then summed. A higher score indicated a greater perceived risk.

We collected information about participants’ demographic characteristics and their professional and training backgrounds. Demographic characteristics included age (years), gender (male vs. female), ethnicity (Kinh, Thai and other), education level (≥ 4 years of medical training vs. lower), and job position (physician/physician assistant vs. other). Professional and training information included years working in the medical field, number of PWUD seen in a month, SUD-related training (≥ 2 courses, 1 course and 0), and HIV-related training (ever vs. never been trained). We collected characteristics at the commune level including the number of ethnicity groups in the CHC’s catchment area and the ART provision status (yes vs. no) of the CHC.

### Statistical analysis

First, we conducted a descriptive analysis of PCPs’ demographic characteristics and their knowledge, attitudes and perceptions related to their confidence in providing methadone treatment. We presented categorical variables by number and percentage for each group and summarized continuous variables by means and standard deviations. All characteristics and scales were compared between PCPs with vs. without experiences in providing methadone treatment using appropriate statistic tests (i.e., chi-squared test for categorical variables and ANOVA test for continuous variables). The methadone experience status of PCP (i.e., with vs. without methadone experiences) was based on their real experience, not the status of their CHC. If a PCP who worked at a CHC without methadone treatment had been involved in methadone programs in the past, he or she was categorized as “having experience with methadone”. We also assessed the Pearson correlation between PCPs’ confidence in providing methadone treatment and potential associated characteristics.

Second, we conducted three linear regression models to explore factors associated with PCP confidence in providing methadone treatment among all participants and then by their methadone experience status. Independent variables were included in the models given their associations with the main outcome (statistically significant level was set at α = 0.05). All models were controlled for background characteristics (age, gender, and ethnicity), and commune-level characteristics (ART provision status and number of ethnicity groups in the CHC’s catchment area). A model of the whole study population examined the difference in confidence between PCPs with experiences in providing methadone treatment and those without. In this model, we also tried to explore potential effect modification of methadone experiences by adding two-way interaction terms with other important PCP characteristics. The two models among subgroups aim to explore factors associated with confidence level in performing methadone tasks among those with experiences in providing methadone treatment and those without. All analyses were performed in SAS software version 9.4 (SAS Institute, Inc., Cary, NC).

### Ethics

The study has been approved by Institutional Review Board (IRB) of University of California, Los Angeles, the United States and Hanoi Medical University, Vietnam.

## Results 

### Socio-demographic and professional characteristics of the study participants

No participants refused to take part in the study. Among the 276 PCPs who participated in the study, 135 (48.9%) were female, 200 (72.5%) were physicians or physician assistants, and 44 (15.9%) had at least 4 years of medical education. 114 PCPs (41.3%) had experiences providing methadone treatment, either as managers or clinicians. The two most popular ethnicity groups were Thai (51.5%) and Kinh (37.7%). Overall, 61.3% had previously had SUD-related training and 72.8% had HIV-related training. While 62.3% of PCPs with methadone experiences had received at least one SUD-related training course, only 37.0% of PCPs without experiences had received training on this topic. The average number of PWUD seen a month was 19.7 (SD 25.7). PCPs with methadone experience saw more PWUD on average (35.8, SD 28.3) compared to those without experience (8.3, SD 15.8) (Table 1).

**Table 1:** Demographic and background characteristics of participants, overall and by MMT experiences (N = 276)

Characteristics	Total (N = 276)	Experienced MMT		*p*
		Yes (n = 114)	No (n = 162)	
Mean (SD)				
Age	34.4 (7.9)	33.8 (8.0)	34.8 (7.9)	.278
Years in medical field	10.4 (7.7)	10.1 (8.2)	10.6 (7.3)	.573
Number of PWUD seen monthly	19.7 (25.7)	35.8 (28.3)	8.3 (15.8)	< .001
N (%)				
Gender				
Male	141 (51.1)	55 (48.2)	86 (53.1)	.428
Female	135 (48.9)	59 (51.8)	76 (46.9)	
Ethnicity				
Kinh	104 (37.7)	53 (46.5)	51 (31.5)	.013
Thai	142 (51.5)	54 (47.4)	88 (54.3)	
Other	30 (10.8)	7 (6.1)	23 (14.2)	
Job position				
Physician/Physician Assistant	200 (72.5)	76 (66.7)	124 (76.5)	.071
Other	76 (27.5)	38 (33.3)	38 (23.5)	
Years of medical education				
≥ 4 years	44 (15.9)	24 (21.1)	20 (12.4)	.052
< 4 years	232 (84.1)	90 (78.9)	142 (87.6)	
SUD-related training				
≥ 2 training	96 (34.8)	71 (62.3)	25 (15.4)	< .001
1 training	73 (26.5)	38 (33.3)	35 (21.6)	
Never	107 (38.7)	5 (4.4)	102 (63.0)	
HIV-related training				
Ever	201 (72.8)	89 (78.1)	112 (69.1)	.101
Never	75 (27.2)	25 (21.9)	50 (30.9)	

PWUD, People who use drugs; SUD, substance use disorder; HIV, human immunodeficiency virus

### Confidence in providing methadone treatment and its related knowledge and perception

Table 2 shows PCP level of confidence in providing methadone treatment by demographic and background characteristics overall and by methadone experience. Although the ethnic distribution were different among PCPs with and without methadone experience, we found no difference in their confidence level in providing methadone treatment. Physicians/physician assistants and other PCPs who had received at least two SUD-related training courses were more confident in providing methadone treatment compared to others (i.e., those were not physicians/physician assistants or had received less training). We found no difference in confidence among PCP by gender, ethnicity, education level, and HIV-related training in the whole population. In general, PCPs with methadone experience were more confident in providing methadone treatment than PCPs with the same characteristics without experience.

**Table 2:** Confidence in providing MMT services by participants’ demographic and background groups, overall and by MMT experiences (N = 276)

Characteristics	Overall (N = 276)		Experienced MMT, Mean (SD)		*p*
	Mean (SD)	*p*	Yes (n = 114)	No (n = 162)	
Gender		.484			
Male	45.5 (12.4)		52.3 (8.4)	41.1 (12.6)	< .001
Female	44.5 (11.7)		48.4 (9.2)	41.4 (12.5)	< .001
Ethnicity		.638			
Kinh	45.8 (11.9)		50.4 (8.8)	40.9 (13.0)	< .001
Thai	44.7 (12.4)		50.3 (9.4)	41.3 (12.8)	< .001
Other	43.6 (10.6)		49.6 (9.1)	41.7 (10.5)	.088
Job position		.050			
Physician/ Physician Assistant	45.9 (12.)		52.6 (7.4)	41.7 (12.5)	< .001
Other	42.7 (11.8)		45.7 (10.2)	39.6 (12.7)	.022
Years of medical education		.939			
≥ 4 years	45.1 (13.6)		53.8 (6.0)	34.7 (12.8)	< .001
< 4 years	45.0 (11.8)		49.4 (9.5)	42.2 (12.2)	< .001
SUD-related training		< .001			
≥ 2 training	48.5 (10.4)		50.5 (9.1)	42.5 (11.5)	< .001
1 training	47.2 (10.1)		50.5 (8.7)	43.7 (10.5)	.004
Never	40.3 (13.2)		45. 8 (10.6)	40.1 (13.3)	.346
HIV-related training		.141			
Ever	45.6 (11.8)		51.1 (8.6)	41.3 (12.2)	< .001
Never	43.2 (12.5)		47.4 (10.0)	41.2 (13.2)	.043

SUD, substance use disorder; HIV, human immunodeficiency virus

Table 3 demonstrated the average score and standard deviation of PCP confidence in providing methadone treatment and, potentially, related knowledge, attitudes and perceptions. The average confidence level was 45.0 (SD 12.0). It was higher among PCPs with methadone experience compared to those without (mean scores 50.3 vs. 41.2, p < 0.001). The participants provided on average 13.2 correct answers (SD = 3.2; range: 0–19) out of 20 questions on methadone knowledge. PCPs with methadone experience, compared to those without, had significantly higher methadone knowledge, higher belief in harm reduction, and higher perceived work-related support. Although PCPs with and without methadone experience were similar in the empathy towards PWUD and perceived risk in working with PWUD, inexperienced professionals exhibited more perceived stigma of working with PWUD (mean scores 12.5 vs. 11.1, p = 0.012).

**Table 3:** Confidence levels in providing MMT services and related scales of participants, overall and by MMT experiences (N = 276)

	Overall, Mean (SD) (N = 276)	Experienced MMT Mean (SD)		*p*
		Yes (n = 114)	No (n = 162)	
MMT knowledge	13.2 (3.2)	15.0 (2.1)	11.9 (3.2)	< .001
Belief in harm reduction	12.9 (1.9)	13.3 (1.9)	12.7 (1.9)	.014
Perceived work-related support	15.5 (4.5)	16.3 (4.1)	14.8 (4.7)	.007
Empathy towards PWUD	76.4 (9.6)	75.5 (8.4)	77.1 (10.4)	.167
Perceived stigma of working with PWUD	11.9 (4.5)	11.1 (4.6)	12.5 (4.4)	.012
Perceived risk of working with PWUD	11.5 (3.8)	11.1 (3.7)	11.8 (3.8)	.110
Confidence in providing MMT services	45.0 (12.0)	50.3 (9.0)	41.2 (12.5)	< .001

PWUD, People who use drugs; MMT, methadone maintenance treatment

### Factors associated with confidence in providing methadone treatment in PCPs overall and by methadone experience

Table 4 presents the results of three regression models on PCP confidence in providing methadone treatment. In the first model that considered all participants, PCP confidence was associated with job position (i.e., physicians/physician assistants vs. others) (β = 3.60, 95% CL 0.85; 6.35), methadone knowledge (β = 0.75, 95% CL 0.30; 1.20), and perceived work-related support (β = 0.66, 95% CL 0.39; 1.93). The main effect of having methadone experience (β = 3.08, 95% CL − 0.72; 6.87) was not associated with confidence in providing methadone treatment. The main effect of having ≥ 4 years of medical education (β =− 5.71, 95% CL − 10.34; − 1.07) and the interaction between experience and years of medical education (β = 8.68, 95% CL 2.21; 15.15) were significantly associated with the main outcome. These results suggested opposite associations between confidence and years of medical education among providers with methadone experience and those without.

**Table 4:** Multi-variable regression analysis on primary care providers’ confidence in providing MMT services, overall and by MMT experiences (N = 276)

	Overall (N = 276)				Experienced MMT (n = 114)				Not experienced MMT (n = 162)		
Variables	β	95% CL	*p*		β	95% CL	*p*		β	95% CL	*p*
Physician/Physician Assistant vs. Other	3.60	0.85; 6.35	.011		5.17	1.95; 8.40	.002		2.52	− 1.90; 6.94	.261
≥ 4 years medical education vs. Lower	− 5.71	− 10.34; − 1.07	.016		2.72	− 1.06; 6.49	.155		− 5.80	− 11.22; − 0.39	.036
Experienced MMT	3.08	− 0.72; 6.87	.111		–	–	–		–	–	–
Experienced MMT *Years of medical education	8.68	2.21; 15.15	.009		–	–	–		–	–	–
SUD-related training											
≥ 2 training	1.11	− 2.48; 4.70	.543		2.92	− 4.41; 10.26	.429		1.35	− 4.15; 6.85	.628
1 training	2.36	− 1.02; 5.73	.170		3.88	− 3.53; 11.29	.230		2.76	− 1.79; 7.32	.232
Never	Ref	–	–		Ref	–	–		Ref	–	–
Number of PWUD seen monthly	0.04	− 0.02; 0.10	.152		0.04	− 0.02; 0.09	.194		0.03	− 0.09; 0.15	.654
MMT knowledge	0.75	0.30; 1.20	.001		0.30	− 0.38; 0.99	.381		0.90	0.29; 1.51	.004
Belief in harm reduction	0.34	− 0.30; 0.98	.302		0.21	− 0.59; 1.00	.606		0.40	− 0.58; 1.38	.419
Perceived work-related support	0.66	0.39; 0.93	< .001		0.71	0.35; 1.08	< .001		0.58	0.19; 0.96	.004

All models adjusted for age, gender, ethnicity of providers, ART status of commune health centers, number of ethnicity group in the catchment area

PWUD, People who use drugs; MMT, methadone maintenance treatment; SUD, substance use disorder

The association between confidence and education levels was negative among PCPs without methadone experience (β = -5.80, 95% CL − 11.22; − 0.39) and positive among experienced PCPs, although not statistically significant (β = 2.72, 95% CL − 1.06; 6.49). Methadone treatment knowledge was associated with confidence among inexperienced PCPs (β = 0.90, 95% CL 0.29; 1.51) but not among the experienced ones (β = 0.30, 95% CL − 0.38; 0.99). In contrast, perceived work-related support was positively associated with confidence among both experienced (β = 0.71, 95% CL 0.35; 1.08) and inexperienced groups (β = 0.58, 95% CL 0.19; 0.96).

## Discussion

This study is among the few that examines PCP confidence in providing methadone treatment in mountainous and rural areas. Given the low availability of specialized substance use treatment in these settings, integrating methadone treatment into CHCs would be the only option to provide treatment to local residents in need [24, 39]. We found differences in confidence level, knowledge, and attitude toward methadone treatment between PCPs who were experienced in methadone treatment and those who were not. We also found several factors associated with the confidence level of PCPs by methadone experience. These findings have important implications for designing interventions and policies to improve health providers’ confidence, and thus, could improve decentralized methadone treatment implementation and quality.

Unsurprisingly, PCPs with experience in methadone treatment reported higher confidence in providing these services and more accurate knowledge about methadone treatment. They also endorsed harm reduction beliefs more often and perceived less stigma from working with PWUD. Stigma and disbelief in harm reduction are challenges to providing methadone treatment in rural primary care settings where strong biases shape PCP willingness provide such treatment [9]. With the differences in SUD-related training and working experiences (i.e., number of PWUD seen monthly), results suggested that training with both theoretical and practicum components could help change PCP beliefs and attitudes toward PWUD and methadone treatment.

Confidence in providing methadone treatment was associated with distinguished factors across different subgroups of PCPs, depending on their methadone experience. While job position was associated with confidence in experienced PCPs, medical education levels played an important role in how confident inexperienced PCPs were. Among PCPs without methadone experience, those with higher education reported a lower confidence level on average. However, among PCPs with methadone experience, those with higher education expressed a higher level of confidence. It may reflect the fact that those with higher education are more aware of the risks associated with methadone prescription (e.g., overdose, interactions with other treatment, etc.) and feel more comfortable gradually with clinical experience [19, 22]. These results emphasize the importance of training and technical support for all professionals working in methadone programs [33]. Our results also suggest interventions to improve the quality of decentralized methadone treatment should be tailored to different subgroups to maximize their impact, especially in the limited resource context of low-and-middle income countries [34].

This study, in line with previous literature, documented the important roles of methadone treatment knowledge and technical support in PCP confidence in methadone treatment [8, 20]. In general, more methadone knowledge is associated with higher confidence in providing treatment. However, in our study, only PCPs without methadone experience felt more confident when they learned more about methadone treatment. Accreditation training and regular technical support would be helpful to boost providers’ confidence to work with drug-using patients as suggested in other studies [1, 4, 10, 15]. Healthcare providers in Vietnam are required to complete an accreditation training on methadone treatment before they can start working in a methadone clinic. Yet some providers in our study had been deployed to methadone programs without this training due to the pressure to expand treatment coverage. The lack of training and continued education in methadone treatment might negatively impact treatment quality. As methadone programs continue to be decentralized at the commune level, we should pay more attention to ensure appropriate training for clinical staff to deliver high quality services.

### Limitations

The findings of this study should be interpreted considering our limitations. While this is the first study exploring factors associated with PCP confidence in providing methadone treatment in rural areas, we were only able to collect data from one province. Moreover, the cross-sectional design prevents us to make causal inferences for the identified associations. However, as we included both urban and mountainous/rural areas of the selected province, the study results would provide valid evidence about the situation of methadone decentralization in Vietnam. Further studies could examine providers’ performance and quality of decentralized treatment from patients’ perspectives. This additional information would provide us with a better understanding of how to improve methadone treatment decentralization.

## Conclusion

Future interventions to improve PCP confidence should be tailored to different subgroups of PCPs with or without methadone experience. Technical knowledge about the treatment is important to prepare new methadone providers for their job. However, providers with treatment experiences might benefit from greater support, such as regular technical assistance throughout their work with PWUD. To ensure the quality of methadone treatment integrated in primary care in remote areas, continued support for PCPs should be a priority.

## Data Availability

The datasets used and/or analysed during the current study are available from the corresponding author on reasonable request.
